# Prenatal stress increases corticosterone levels in offspring by impairing placental glucocorticoid barrier function

**DOI:** 10.1371/journal.pone.0313705

**Published:** 2025-07-18

**Authors:** Can Liu, Hongya Liu, Hongyu Li, Deguang Yang, Ye Li, Rui Wang, Jiashu Zhu, Shuqin Ma, Suzhen Guan

**Affiliations:** 1 School of Public Health, Ningxia Medical University, Yinchuan, Ningxia, China; 2 Key Laboratory of Environmental Factors and Chronic Disease Control, Yinchuan, Ningxia, China; 3 Chongqing Medical University, Chongqing, China; 4 General Hospital of Ningxia Medical University, Yinchuan, Ningxia, China; Royal College of Surgeons in Ireland, IRELAND

## Abstract

This study aimed to investigate the association between prenatal stress (PS) and corticosterone levels, and its influence on DNA methylation of genes related to the placental glucocorticoid (GC) barrier, including 11β-HSD2, ABCB1 (P-gp), NR3C1, and FKBP5. The PS model was established through chronic unpredictable mild stress (CUMS). DNA methylation of GC-related genes was analyzed by reduced representation bisulfite sequencing (RRBS), and the results were confirmed using MethylTarget™ sequencing. The mRNA and protein expression levels of these genes were detected through qRT-PCR and Western blotting, respectively. Plasma corticosterone levels were elevated in pregnant female rats exposed to PS conditions and their offspring. Compared to the offspring of the prenatal control (OPC) group, the offspring of the prenatal stress (OPS) group exhibited down-regulation in both mRNA and protein expression of DNA methyltransferases (DNMT 3A and DNMT 3B), while up-regulation was observed in the expression of DNMT1. RRBS analyses identified ABCB1 and FKBP5 as hypermethylated genes, including a total of 43 differentially methylated sites (DMS) and 2 differentially methylated regions (DMR). MethylTarget™ sequencing further confirmed 15 differentially methylated CpG sites in these genes. This study provides preliminary evidence that PS disrupts the placental GC barrier through abnormal gene expression caused by hypermethylation of GC-related genes, resulting in elevated corticosterone levels in offspring and affecting their growth and development.

## 1. Introduction

Pregnancy, although widely regarded as a “natural” physiological process, is also a significant source of stress for women during gestation. Pregnant women may be exposed to various stressors, including environmental toxins, family conflicts, work-related stress, and psychological trauma, which can disrupt their mental health. Numerous epidemiological and case-control studies have shown that maternal psychological and social stress during pregnancy (prenatal stress, PS) not only increases the mother’s own health risks [1,2] but is also closely associated with adverse pregnancy outcomes, such as miscarriage, preterm birth, and low birth weight [3–6]. Additionally, anxiety induced by prenatal stress has been identified as a significant risk factor for emotional disorders and depressive behaviors in offspring [7].

According to Barker’s “Developmental Origins of Health and Disease (DOHaD)” hypothesis [8,9], prenatal stress may lead to cognitive and mental disorders in offspring by altering maternal physiological and metabolic responses. Recent studies suggest that epigenetic modifications induced by PS may underlie structural and physiological abnormalities in offspring [10–13]. However, although there is a clear link between PS exposure and adverse health outcomes in offspring, the underlying mechanisms of PS remain unclear.

Currently, significant attention has been focused on the relationship between stress exposure during pregnancy and adverse health outcomes. The placenta, which acts as a barrier protecting the fetus from high maternal glucocorticoids (cortisol or corticosterone), may have its function compromised under PS conditions. Studies have shown that under PS, the hypothalamic-pituitary-adrenal (HPA) axis is persistently activated, leading to excessive secretion of glucocorticoids (GC) [14]. Elevated maternal cortisol levels may disrupt the placental GC barrier, exposing the fetus to excessive cortisol and thereby causing dysfunction in multiple systems, including the nervous, endocrine, and immune systems [15]. Notably, impaired integrity of the placental GC barrier is closely associated with intrauterine growth retardation and an increased risk of chronic diseases in offspring [16].

Recent research has found that PS regulates the expression of placental GC barrier-related genes through epigenetic mechanisms, such as DNA methylation, thereby affecting offspring development and health [17]. Among these, 11β-hydroxysteroid dehydrogenase type 2 (11β-HSD2) and ABCB1 protect the fetus from high glucocorticoid levels by metabolizing and transporting cortisol, respectively [18–20]. FKBP5 modulates the activity of the glucocorticoid receptor (GR), influencing glucocorticoid signaling, while NR3C1 encodes GR and mediates the biological effects of glucocorticoids [21,22]. Studies have shown that PS-induced changes in NR3C1 methylation are associated with impaired self-regulation in newborns [23,24] and are linked to maternal depression and adverse experiences [25,26]. Although the connection between maternal care and offspring NR3C1 methylation has been established, the comprehensive impact of PS on the epigenetic changes of placental GC barrier-related genes requires further investigation.

In this study, we utilized the chronic unpredictable mild stress (CUMS) model to investigate the effects of prenatal stress (PS) on DNA methylation of placental glucocorticoid (GC) barrier-related genes (11β-HSD2, ABCB1, NR3C1, and FKBP5) in pregnant rats and their association with corticosterone. Based on previous research findings, we propose the following hypothesis: PS regulates the expression of placental GC barrier-related genes through abnormal DNA methylation, leading to elevated corticosterone levels in both the mother and fetus, thereby providing a novel epigenetic perspective for understanding the impact of PS on offspring health.

## 2 Materials and methods

### 2.1 Chemicals and reagents

The Iodine-131 cortisol radioimmunoassay kit was obtained from the Beijing North Institute of Biotechnology. The RNA extraction kit, first strand cDNA synthesis kit, and real-time PCR reaction kit were purchased from Beijing Tiangen Biochemical Technology Co., Ltd. The RIPA lysis solution was from Nanjing KGI Biotechnology Co., Ltd. The whole protein extraction kit and bicinchoninic acid (BCA) protein content detection kit was utilized. Sodium dodecyl sulfate-polyacrylamide gel electrophoresis (SDS-PAGE) gel preparation kit and substrate chemiluminescence (electro-chemo-luminescence, ECL) kit were purchased from Nanjing KGI Biotechnology Co., Ltd.

Primary antibodies including anti-β-actin (AF7018), anti-DNA methyltransferases (DNMT) 3A (DF7226), anti-DNMT 3B (AF5493), anti-DNMT1 (DF7376) and Goat-Rabbit IgG (S0001) were purchased from Affinity Technology Co., Ltd. Anti-11β-HSD2 antibody was acquired from Novus Technology Co., Ltd. (NBP1–39478). The anti-ABCB1 (ab170904) and anti-NR3C1 (ab183127) were purchased from Abcam Technology Co., Ltd. The anti-FKBP5 (00083356) was purchased from Proteintech Technology Co., Ltd.

### 2.2 CUMS procedure

Sixteen healthy adult female Sprague-Dawley (SD) rats, weighing (200 ± 20) g, and twelve male Sprague-Dawley (SD) rats, weighing (220 ± 20) g, were obtained from the Experimental Animal Center of Ningxia Medical University, with an experimental animal certificate number SYXK(Ning)2020–0001. All experimental procedures were approved by the Animal Care and Use Committee of Ningxia Medical University (Approval No. 2022-N107) and strictly adhered to the ‘3R principles’ (Replacement, Reduction, Refinement) in accordance with the National Institutes of Health (NIH) Guide for the Care and Use of Laboratory Animals (IACUC-NYLAC-2020068).. Rats were group-housed in cages under controlled temperature conditions (22 ~ 24°C), with a 12-hour light/dark cycle.

Female rats were randomly assigned to either a prenatal stress (PS) group or a prenatal control (PC) group, with eight rats in each group. Male rats were randomly divided into a control mating group (four rats) or a stress mating group (eight rats). Mating for the PS group commenced on day 7 after exposure to chronic unpredictable mild stress. Before gestation, female rats from both groups mated with males from their respective mating groups. Pregnancy was determined by checking the vaginal smear for the presence of a pubic plug, and the birth date of the offspring was designated as gestational day 0 (GD 0), subsequently, male and female rats were separated.

After gestation, the rats were returned to their original breeding environment. During mating, the PS group rats experienced continuous stress stimulation. To induce chronic stress conditions, we employed the chronic unpredictable mild stress (CUMS) procedure based on a previously described method with minor modifications and additions [2,27]. The PS group rats were exposed to one randomly selected stressor out of seven different types daily for four weeks. These stressors included crowded environments for 24 hours, physical restraint (2 hours), filthy cages for 24 hours (60–70% humidity, 12 hours), hot water swimming at 45°C for 5 minutes, rocking cages for 30 minutes, ice bath at 4°C for 5 minutes, and tail squeezing for 2 minutes. Each day, one of these seven different stressors was randomly administered. The duration of the daily exposure to the twenty-four-hour stressors was from morning at 10:00 until the next morning at 10:00; all other stressors occurred between morning at 10:00 and noon. Throughout this process, stressed rats were temporarily moved to another room with identical light intensity and temperature before being returned to the main room following stimulation.

### 2.3 Placental sampling

After 18 days of gestation, six female rats (three randomly chosen from the PS group and three from the PC group) were anesthetized with isoflurane, and the uterus tissues were promptly removed. Rats were anesthetized with 3–5% isoflurane in oxygen for induction, then maintained on 1–2% isoflurane via a nose cone. Anesthesia depth was verified by lack of response to toe pinch. To minimize postoperative pain, meloxicam (2 mg/kg) was administered subcutaneously after surgery. Animals were monitored for pain or distress during experiments; any severely distressed animal would be euthanized immediately, though none occurred.

Following tissue collection, rats were euthanized by cervical dislocation under deep anesthesia, consistent with the AVMA Guidelines for the Euthanasia of Animals (2020 edition). Death was confirmed by cessation of heartbeat and respiration. The uterus-embryo mixture was pre-cooled in PBS solution, followed by removal of the uterus and fetal membrane tissues, and subsequent separation of the placenta. Three placental tissues from each group were immediately frozen in liquid nitrogen stored at −80°C for subsequent examination.

### 2.4 Allocation of the offspring into groups

On the day of birth, the dams and their pups were kept together until postnatal day (PND) 21, when weaning occurred and male and female pups were separated. After ten pregnant rats gave birth, two offspring (one male and one female) were randomly selected from each litter. These offspring from the PC group and PS group were then assigned to either the offspring of the prenatal control (OPC) group (n = 10; 5 males vs 5 females) or the offspring of the prenatal stress (OPS) group (n = 10; 5 males vs 5 females). All experimental offspring were housed in the same room with free access to water and food. Body weight were measured, and blood samples were collected from the inner canthal vein on PND 28 and PND42. A schematic diagram of the experimental procedure is shown in S1 Fig.

### 2.5 Measurement of selected indicators

#### 2.5.1 Measurement of plasma corticosterone concentration.

The successful establishment of the CUMS model was confirmed by measuring the plasma corticosterone concentration in female rats at different time points during stress. Blood samples (1 mL) were collected from the inner canthus vein of rats using heparin-anticoagulant tubes on the day before the first stress (baseline), as well as on days 1, 7, 14, 21, and 28 during the stress period. The plasma was separated by centrifugation at 3000 rpm for 15 minutes at 4 °C and stored at −80°C. Corticosterone levels were measured using a ^131^I cortisol radioimmunoassay (RIA) kit according to the manufacturer’s instructions. The intra-assay variability of RIA ranged from 3.2% to 4.7%. Plasma corticosterone concentrations were calculated using a conversion formula: corticosterone concentration = cortisol concentration ×50 [28].

#### 2.5.2 Preparation of RRBS library of placenta.

Genomic DNA from placental samples of two offspring groups (three placental samples in the OPC group and three placental samplings in the OPS group) was extracted using a magnetic universal genomic DNA Kit following the manufacturer’s protocol. After that, reduced representation bisulfite sequencing (RRBS) library preparation was performed using Acegen Rapid RRBS Library Prep Kit according to the manufacturer’s instructions [29].

#### 2.5.3 Illumina Hiseq sequencing platform.

RRBS sequencing was conducted at Biomarker Technologies Co.,Ltd. Genomic DNA (100 ng) was digested with MspI, end-repaired, tailed with dA nucleotides, and ligated to adapters modified with methylcytosine residues. After digestion, genomic DNA purification was carried out using Agencourt® AMPure® XP Nucleic Acid Purification Kit, followed by bisulfite treatment utilizing ZYMO EZ DNA Methylation-Gold Kit. Illumina double index primers with an eight-base pair sequence were used for PCR amplification, which consisted of twelve cycles. For the quality analysis of libraries, an Agilent 2100 bioanalyzer (Agilent Technologies, Santa Clara, CA, USA) was used. Quantification of DNA fragments ranging from 25 bp to12,000 bp was carried out using ABI StepOnePlus real-time PCR system (Thermo Fisher Scientific). The fragments were sequenced using a 150 × 2 paired terminal sequencing scheme on the Illumina NovaSeq 6000 analyzer (Illumina HiSeq sequencing platform) to obtain the sequencing results. Subsequently, methylation analysis was performed on the promoter and CpG island region by detecting the proportion of CG, CHG, and CHH in the methylated C base, the average methylation level of the C base, and the distribution of different types of methylation. Regional methylation profiles were calculated based on methylation levels and CpG density for specific regions. CpG density was defined for each CpG site within a 200 bp window. Differential Methylation Region (DMR) analysis was conducted using a sliding-window method to calculate the methylation difference between placental samples from the OPC and OPS groups [29,30].

#### 2.5.4 Validation of DNA methylation status and expression levels of target genes.

The DNA methylation status of the ABCB1 and FKBP5 introns was analyzed using the MethylTarget™ assay (targeted bisulfite sequencing) [30,31]. Target genes with the greatest differences in DNA methylation were subjected to further analysis (S1 Table). Six placentas from the OPC group and six placentas from the OPS group was used to validate the results of RRBS and to detect the expression of these differentially methylated genes. Sample acquisition, DNA extraction, and preservation were performed as mentioned above.

Firstly, 40 ng of genomic DNA was subjected to bisulfite conversion using the EZ DNA Methylation-Gold™ kit (Zymo Research) according to the manufacturer’s instructions. Then, PCR amplification was carried out to amplify the targeted DNA sequences. The resulting products were sequenced on an Illumina MiSeq benchtop sequencer. The primer sequences for the target regions within FKBP5 and ABCB1 loci are listed in Table 1 below. FastQC software was used for the quality control assessment of the sequencing reads. The filtered reads were subjected to read recalibration with USEARCH and then mapped to the genome by Blast.

**Table 1:** Primers for MethylTarget^TM^ methylation sequencing and qRT-PCR analysis.

Gene name	Primer Forward	Primer Reverse
*FKBP5_01*	TAGTGAGGGAGTATTTGAGAGTATTGTT	AACCTAAAATATTAAAACATTCRAAACTAACC
*FKBP5_02*	AGTAAGTYGGTTAGTTATTAYGTTAGGATTTT	AACCTCCCTAACCTACAATTCTAATCAA
*FKBP5_03*	AAAGTATTGAGTAAGAAGGTAGTAGAAGGAG	CTTACCATTAACTTCCAAAATCACAA
*FKBP5_04*	AGGGATGGTTGTGTATTTAGGTTTTT	TACCACAACCAAAATCCACCAC
*FKBP5_05*	TATGTTGGGTAAAGTTGGGTTTG	TATCTCAACTTTAATTTCCTCAAAACA
*FKBP5_06*	GGTATAGTAGGGGTTGGGGATTT	ACAAAATCTCATACCACTAAAACTAACCTT
*FKBP5_07*	AAATGGAAAGGAGATAAAGAAGTTTG	TCTCCACACAACCCTACTCTACAAA
*FKBP5_08*	AGTTGGTTGGTTTGGTTAGTGTGAA	AACCTCACCCACTTCTTCATCTTC
*abcb1a_09*	TGTTATAGTTGTGTAGTTTGGGTGTTT	TCCAAAAATATCAACCAAACATACCAT
*abcb1a_10*	ATGTGTTAGTGTTTTAGGGGTTGAGTTT	CCAAATAAAATTTCAAACCTTCACTCA
*abcb1a_11*	TTGGAAGTTAGGTTGTTTAAAATATTGAGA	AATAAAATCATAAACATTAACCTCTTTAACAAC
*abcb1a_12*	TTTGAAGTATTGTTGAGTAAGGAGAAGGTA	ACTACACTCCTAACAACTAACCCTAACA
*abcb1a_13*	TTTGTTGTTATGTAGTTGTGTGTTTTT	CTTAACTAACCCTCAATCCAAACATAA
*abcb1a_14*	TTGYGTTGTAGGTTGTTGGTAGTTTGTT	ATTAACCACAACTAATAACRTACAAACCTA
*abcb1a_15*	TTTGGTTATGTATTTAGGAGTATAGTAGGTTTTT	CCCCTTCCCCATCCTACC
*abcb1a_16*	ATGTTTATAGGTGGAATTAAGTGTGTTTT	TTCTAAATACCCATATAACATCTCACACC
*abcb1a_17*	TGGATTTTTATTTGTATTGTTGTTGTAG	ACTCCTTCCCTAACTCCCTATTTTT
*abcb1a_18*	TAGGGTTTGGGAGTAGAGAGTTGTT	AAAAATCCAATAAATCCCACAAAAAC
*abcb1a_19*	GAAAGTTGTTAAGGAAGTTAATGTTTATG	TCTCAATTCACRTCTCRAACTAAAAA
*abcb1a_20*	TTTTTAGTGTAGATATAGGGGAGTGTTG	AACCAACCTATCTCCTAATTCATAATAACA
*abcb1a_21*	TTAAGTGTTGGGATTGTTATGTTTG	AACAATCCRTCTAAAACTACAACCACTAA
*abcb1a_22*	AAGGTTAATATTGAAGGGGTTGTATTT	CTCAAAACCCCACAAAACCT
*abcb1a_23*	ATGTGGGTTTTGGGTGTGAA	AAACCCACCATAAATCCTTCCTAA
*abcb1a_24*	GTTTTTAAGATAGAATTTATTTGAGGTTAGAT	AAACCAATCCTATATAATCATCCCCATT
*abcb1a_25*	AAYGAGGTTAAATAAGTTTGAGTTTGTATT	CTTACATACCATACATTCTAAAACAAAACA
*abcb1a_26*	TTAGAGAGAAGGATTGGTTAGAAAATAGTTA	CTAACTTAAACATAACACRAAATACTCACTCTA
*DNMT 3A*	CGTCACACAGAAGCATATCCAGGAG	CAGGAGGCGGTAGAACTCAAAGAAG
*DNMT 3B*	GATGGAGATGGTGAAGCGGATGATG	AGGCTGGAGATACTGTTGCTGTTTC
*DNMT1*	TGTTCCTCCTTCTGCCATCAATGTG	CATCGTCCTTAGCGTCGTCGTAAC
*11β-HSD2*	CCTCTGGACCTCTCCTCTGCTTC	GCCACATCTCACGCTAAACTCTCC
*abcb1a*	GAAGAAGACCTTAACGGAAGAGCAGAC	GCGAAACATTGTGAGCACACTGAC
*abcb1b*	CTCGCTGCTATCATCCACGGAAC	CGCTGACGGTCTGTGTACTGTTG
*NR3C1*	CACATCTCACCCGCACCGATTG	TTGGACAAACACGGATGCCTGAC
*FKBP5*	TGGTGGGCATGGCAGGGATC	GCTTGTGTCAGGCGAGTCAGTG
*β-actin*	TGTCACCAACTGGGACGATA	GGGGTGTTGAAGGTCTCAAA

#### 2.5.5 Quantitative real-time PCR (qRT-PCR).

The RNA was extracted from isolated placental tissues according to the protocol of the total RNA extraction kit. The extracted RNA was quantified using a nucleic acid-protein quantitative instrument, and samples with an A260/280 ratio between 1.8 and 2.0 were selected for further analysis. A two-step Real-time Quantitative PCR (RT-qPCR) assay was performed to analyze gene expression levels of DNA methyltransferases (DNMT)3A, DNMT3B, DNMT1, 11β-HSD2, abcb1a, abcb1b, NR3C1, and FKBP5. The cDNA was synthesized from total RNA derived from placental tissue (1000ng) in a total volume of 20 µL using a cDNA synthesis kit. PCR reactions were carried out using primers (listed in Table 1) and SuperReal PreMix Plus(SYBR Green) reagent under specific conditions: a pre-denaturation step at 95°C for 15 minutes, followed by 40 cycles of denaturation at 95°C for 10 seconds, annealing at 55°C for 20 seconds, and extension at 70°C for 30 seconds. Finally, the relative expression level of the target gene was calculated using the 2^-ΔΔCt^ method.

#### 2.5.6 Western Blot.

The placental tissue was subjected to protein extraction using a cold Radioimmunoprecipitation assay (RIPA) lysis buffer system. The protein concentration of the samples was determined using a BCA assay kit. Equal amounts of protein were separated by 10% SDS-PAGE and subsequently transferred onto a PVDF microporous membrane. Following blocking with 5% skim milk, the membranes were incubated overnight at 4°C with antibodies of appropriate concentrations, including anti-DNMT1 (1:1000), anti-DNMT3A (1:1000), anti-DNMT3B (1:1000), anti-11β-HSD2 (1:2000), anti-ABCB1 (1:2000), anti-NR3C1 (1:2000), anti-FKBP5 (1:2000), or anti-β-actin antibody (1:2000). Afterward, secondary antibodies were applied at room temperature for 1 hour. Visualization of the blots was achieved using ECL Plus detection reagent from Jiangsu Kaiji Biotechnology Co., LTD.

### 2.6 Statistical analysis

Statistical analysis was performed on all data using the Statistical Package for the Social Sciences (SPSS) version 26.0, while graph construction utilized GraphPad Prism version 8.0. A significance level of 0.05 was adopted for all statistical tests. For the differences in plasma corticosterone levels during prenatal stages, repeated measurements analysis of variance was employed to assess the overall effects of groups and time points, and Student’s t-test was used for multiple comparisons at different time points. All data of plasma corticosterone levels are represented as Mean ± SD. For RT-PCR and Western blotting data, Student’s t-test was used to compare the two offspring groups, with a significance level of 0.05. The data obtained from RT – PCR and Western blotting are also represented as Mean ± SD.

## 3 Results

### 3.1 PS increases prenatal plasma corticosterone levels

**Fig 1:**
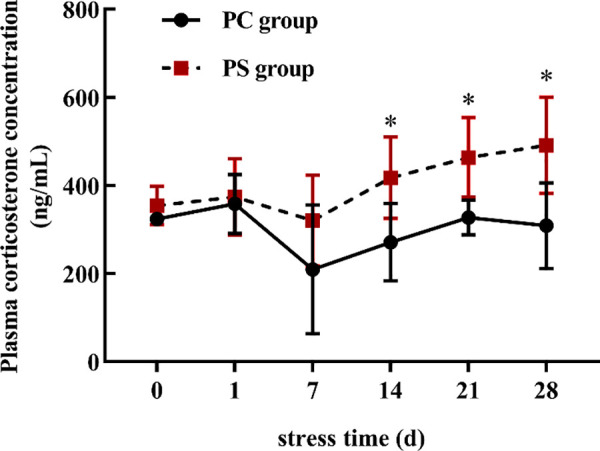
Plasma corticosterone concentration in the PC and PS group. This figure presents the comparison of plasma corticosterone concentrations between the PC (control) group and the PS (experimental) group at different stress time. Data are expressed as mean± SD (N = 6 per group). Statistically significant differences are indicated by *, *P* < 0.05.

Repeated-measures ANOVA results showed that chronic stress had a significant effect on prenatal corticosterone concentration (*F (1,10)* =7.632, *P* = 0.020), and the concentration of corticosterone in the PS group changed with the increase of stress duration ((*F (5, 50)* =6.871, *P* = 0.001). In addition, there was a significant interaction between stressors and time (*F (5, 50)* =3.141, *P* = 0.015). The plasma corticosterone concentrations in the PS group were higher than that in the PC group on days 14, 21, and 28 of stress (*t*_*14*_ *(10)* =−2.811, *P* = 0.018; *t*_*21*_ *(10)* =−3.398, *P* = 0.007; *t*_*28*_ *(10)* =−3.060, *P* = 0.012). The results suggest that the PS group was stressed during pregnancy. Plasma corticosterone concentrations were not statistically significant between the PC and PS groups at baseline examination *(t (10) =−1.607, P = 0.139)* and on day one after exposure to stress *(t (10) =−0.359, P = 0.727)* (Fig 1).

### 3.2 Effects of PS on offspring body weight and plasma corticosterone

**Fig 2:**
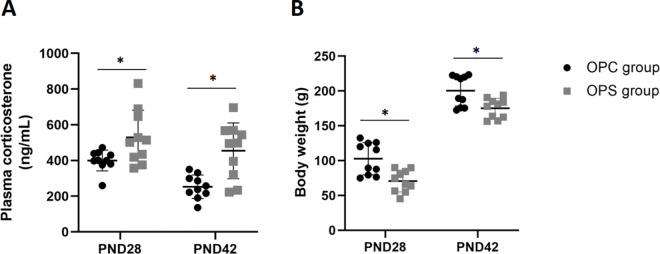
Effects of PS on offspring’s body weight and plasma corticosterone. **(A)** Plasma corticosterone concentrations in offspring on postnatal day 28 (PND28) and postnatal day 42 (PND42). **(B)** Body weight in offspring on PND28 and PND42. Data are represented as mean ± SD (N = 10 per group). *Statistically significant difference compared with the OPC group (*P* < 0.05).

Repeated measures analysis of variance showed significant differences in corticosterone concentrations between the OPS and the OPC group (*F (1,18)* =20.588, *P* = 0.000). Corticosterone concentrations in the OPS group changed with increasing birth time (*F (1,18)* =8.783, *P* = 0.008). No significant difference was found in the interaction between the treatment groups and time (*F (1,18)* =0.903, *P* = 0.355). The plasma corticosterone concentrations in the OPS group were higher than that in the OPC group at PND28 (*t (18)* =−2.537, *P* = 0.027) and PND42 (*t (18)* =−3.764, *P* = 0.003) (Fig 2A). The results suggest that the OPS group was in a state of high plasma corticosterone level.

Repeated measures ANOVA of the two groups showed that PS had a significant effect on offspring body weight. (*F (1,18)* =14.384, *P* = 0.001), and there was a significant difference between time and body weight, but not between interaction and body weight (Time: *F (1,18)* =641.390, *P* = 0.000; Interaction: *F (1,18)* = 0.750, *P* = 0.398). The body weight in the OPS group was lower than that in the OPC group at PND28(*t (18)* =3.645, *P* = 0.002) and PND42 (*t (18)* =3.044, *P* = 0.008) (Fig 2B).

### 3.3 Effects of PS on DNA methyltransferase (DNMT)

**Fig 3:**
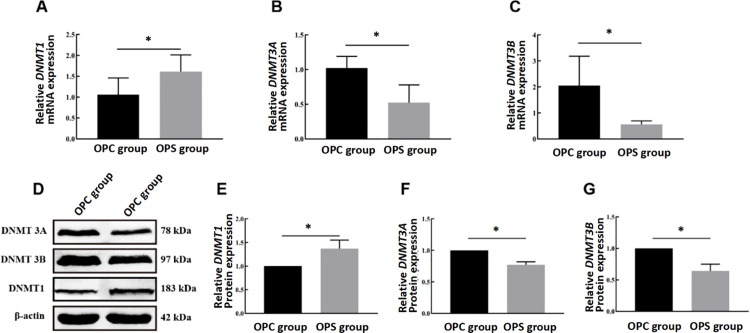
Effects of PS on DNMTs in the placenta. **(A-C)** Relative mRNA expression, (D) representative western blot images, and (E-G) statistical results of relative protein expression of DNMT1, DNMT3A, and DNMT3B. Three biological replicates were performed. All data are shown as mean ± SD (N = 3 per group). *Statistically significant difference compared with the OPC group (P < 0.05).

As shown in Fig 3, the mRNA expression of *DNMT 3A*, *DNMT 3B*, and *DNMT1* was different between the two offspring groups in the comparison of gene expression levels, as shown in Fig 3A ~ C (all *P* < 0.05). Compared with the OPC group, the mRNA levels of *DNMT 3A* (*t (10)* =3.997, *P* = 0.003, Fig 3B) and *DNMT 3B* (*t (10)* =3.210, *P* = 0.009, Fig 3C) were markedly down-regulated and the mRNA expression of *DNMT1* (*t (10)* =−2.401, *P* = 0.037, Fig 3A) was up-regulated in the PS group. The protein levels of DNMT 3A (*t (4)* =8.443, *P* = 0.001, Fig 3D, F) and DNMT 3B (*t (4)* =5.610, *P* = 0.005, Fig 3D, G) were down-regulated in the OPS group, while the protein levels of DNMT1 were up-regulated (*t (4)* =−3.601, *P* = 0.023, Fig 3D, E). Overall, it is shown that PS induces changes in placental DNMTs in the offspring.

### 3.4 DNA methylation status in placenta

RRBS was performed using placental tissues to identify differentially methylated CpG sites and regions. After quality control and data analysis, 222,119 differentially methylated sites (DMS) and 6905 DMRs were identified between the two groups. Compared with the OPC group, 4055 DMRs were highly methylated in the OPS group, and 2850 were low methylation. The analysis revealed that out of the total sites, 110752 were annotated with gene names, while 59778 exhibited hypermethylation and 50974 displayed hypomethylation. Table 2 shows the DMR and gene numbers in differentially methylated genes between the OPC and OPS groups.

**Table 2:** Statistics of DMR in all differentially methylated gene.

Different methylation region	Gene numbers
Promoter	261
5’UTR	60
Exon	1219
3’UTR	121
intron	1648
Distal Intergenic	3509
Downstream	87
Total	6905

Among all the genes with identified DMRs, two genes were associated with placental GC barrier function: ABCB1 and FKBP5. ABCB1 has a total of 32 DMS compared to the OPC group. In the OPS group, 8 sites were hypermethylated and 5 sites were hypomethylated in the exon, and 12 sites were hypermethylated and 7 sites were hypomethylated in an intron. These DMS formed two DMRs, located in the exon and intron regions, both with high methylation levels. For FKBP5, total of 11 DMS were identified (all *P* < 0.05), with 5 sites hypermethylated and 3 sites hypomethylated in the intron, and 1 site was hypermethylated upstream and 2 were hypermethylated downstream. The detailed methylation data of these two genes are shown in Table 3.

**Table 3:** Statistics of DMS and DMR in placental GC barrier-related genes FKBP5 and abcb1a.

Gene	Position	Meth_direction	*P* value	Annotation
*FKBP5*	7975113	strongHyper	0.000388	downstream
*FKBP5*	7975116	strongHyper	0.00181	downstream
*FKBP5*	7977241	strongHypo	0.00809	intron
*FKBP5*	7977273	strongHyper	0.00441	intron
*FKBP5*	7978647	strongHypo	0.000072	intron
*FKBP5*	7991860	strongHypo	0.000641	intron
*FKBP5*	7991871	strongHypo	0.00288	intron
*FKBP5*	7992251	strongHyper	0.00125	intron
*FKBP5*	8000100	strongHypo	0.000573	intron
*FKBP5*	8002228	strongHyper	0.000295	intron
*FKBP5*	8019250	strongHyper	0.00239	upstream
*abcb1a*	22140561	strongHyper	0.00356	intron
*abcb1a*	22140572	strongHyper	0.00524	intron
*abcb1a*	22141030	strongHyper	0.00142	exon
*abcb1a*	22141031	strongHyper	0.000573	exon
*abcb1a*	22141043	strongHyper	0.0057	exon
*abcb1a*	22141058	strongHyper	0.000483	exon
*abcb1a*	22141069	strongHyper	0.000151	exon
*abcb1a*	22141070	strongHyper	0.000373	exon
*abcb1a*	22165829	strongHypo	0.0000386	exon
*abcb1a*	22181973	strongHyper	0.000137	intron
*abcb1a*	22181974	strongHyper	0.000138	intron
*abcb1a*	22182070	strongHyper	0.00111	intron
*abcb1a*	22182101	strongHyper	0.00212	intron
*abcb1a*	22192599	strongHypo	0.00392	intron
*abcb1a*	22222671	strongHypo	0.00295	intron
*abcb1a*	22222675	strongHypo	0.00000464	intron
*abcb1a*	22234557	strongHyper	0.00000554	intron
*abcb1a*	22234706	strongHyper	0.0022	intron
*abcb1a*	22255279	strongHyper	0.00128	intron
*abcb1a*	22279931	strongHypo	0.00478	intron
*abcb1a*	22297010	strongHyper	0.0073	exon
*abcb1a*	22297022	strongHypo	0.00601	exon
*abcb1a*	22304484	strongHyper	0.00327	intron
*abcb1a*	22304577	strongHyper	0.00623	intron
*abcb1a*	22313828	strongHypo	0.00286	intron
*abcb1a*	22320455	strongHyper	0.00229	intron
*abcb1a*	22322568	strongHypo	0.00142	intron
*abcb1a*	22322825	strongHypo	0.000596	intron
*abcb1a*	22397453	strongHypo	0.00429	exon
*abcb1a*	22397481	strongHypo	0.000487	exon
*abcb1a*	22397482	strongHyper	0.0000549	exon
*abcb1a*	22397592	strongHypo	0.0018	exon
*abcb1a*	22141030 ~ 22141072 (43 bp)	strongHyper	0.00000000156	Exon (exon 25 of 28)
*abcb1a*	22181973 ~ 22182103 (131 bp)	strongHyper	0.000000000029	Intron (intron 27 of 27)

### 3.5 PS lead to hypermethylation of GC barrier-related genes

To investigate whether the placental GC barrier-related genes ABCB1 and FKBP5 gene exhibit differential regulation of gene expression between the OPC group and the OPS group, their DNA methylation levels were further confirmed by Methyltarget sequencing.

A total of 12 placental samples, with six in the OPC group and six in the OPS group, were utilized for validation experiments. The validation results (displayed in Table 4) demonstrate that, ABCB1 in the OPS group had 15 differentially methylated CpG sites compared to the OPC group, and three significantly hypermethylated DMRs (abcb1a DMR04, abcb1a DMR05 and abcb1a DMR17), FKBP5 in the OPS group had 15 differentially methylated CpG sites, along with two significantly hypermethylated DMRs (FKBP5 DMR3 and FKBP5 DMR4).

**Table 4:** Differences of methylation levels in specific genes CpG site between different groups.

Target	Position	Chr	Genome	Meth_direction	*t-*value	*P*-value	OPS $\left(\bar{x}\pm s\right)$	OPC $\left(\bar{x}\pm s\right)$
FKBP5 DMR2	161	20	7977242	strongHyper	2.567	0.047	0.755 ± 0.008	0.707 ± 0.045
	201	20	7977202	strongHyper	3.278	0.014	0.939 ± 0.005	0.922 ± 0.012
	230	20	7977173	strongHyper	5.425	0.000	0.874 ± 0.008	0.841 ± 0.012
FKBP5 DMR3 (17 bp)	32	20	7978764	strongHyper	2.810	0.019	0.596 ± 0.075	0.485 ± 0.060
	36	20	7978760	strongHyper	3.113	0.016	0.509 ± 0.082	0.392 ± 0.042
	48	20	7978748	strongHyper	3.003	0.018	0.450 ± 0.078	0.342 ± 0.042
FKBP5 DMR4 (60 bp)	130	20	7978666	strongHyper	2.738	0.022	0.361 ± 0.045	0.282 ± 0.054
	149	20	7978647	strongHyper	2.482	0.037	0.428 ± 0.049	0.335 ± 0.078
	178	20	7991872	strongHyper	7.129	0.000	0.880 ± 0.014	0.831 ± 0.010
	189	20	7991861	strongHyper	2.996	0.028	0.884 ± 0.007	0.835 ± 0.040
FKBP5 DMR5	63	20	7992159	strongHyper	3.353	0.010	0.683 ± 0.023	0.623 ± 0.038
	200	20	7992296	strongHyper	2.247	0.052	0.392 ± 0.036	0.333 ± 0.054
FKBP5 DMR6	54	20	8000070	strongHyper	3.700	0.011	0.959 ± 0.002	0.947 ± 0.007
FKBP5 DMR7	48	20	8002300	strongHyper	1.934	0.092	0.818 ± 0.015	0.791 ± 0.031
	81	20	8002267	strongHyper	3.414	0.007	0.936 ± 0.008	0.920 ± 0.008
	156	20	8002192	strongHyper	2.409	0.050	0.701 ± 0.652	0.604 ± 0.085
abcb1a DMR04 (98 bp)	91	4	22181973	strongHyper	2.639	0.030	0.099 ± 0.038	0.052 ± 0.021
	188	4	22182070	strongHyper	2.554	0.032	0.091 ± 0.027	0.057 ± 0.018
abcb1a DMR05 (69 bp)	74	4	22182070	strongHyper	2.907	0.018	0.088 ± 0.030	0.045 ± 0.020
	141	4	22182137	strongHyper	2.272	0.046	0.170 ± 0.047	0.109 ± 0.046
	156	4	22182152	strongHyper	2.872	0.017	0.260 ± 0.062	0.165 ± 0.052
	209	4	22182205	strongHyper	2.688	0.031	0.079 ± 0.034	0.038 ± 0.016
abcb1a DMR06	49	4	22192666	strongHyper	2.743	0.023	0.013 ± 0.002	0.010 ± 0.002
	164	4	22192551	strongHypo	−3.106	0.012	0.010 ± 0.003	0.014 ± 0.002
abcb1a DMR10	76	4	22255365	strongHypo	−13.949	0.000	0.056 ± 0.091	0.671 ± 0.0581
abcb1a DMR13	58	4	22304610	strongHyper	2.369	0.050	0.420 ± 0.034	0.342 ± 0.074
abcb1aDDMR14	35	4	22313874	strongHyper	2.573	0.041	0.163 ± 0.022	0.139 ± 0.008
abcb1a_DMR15	55	4	22320554	strongHyper	4.918	0.001	0.354 ± 0.033	0.211 ± 0.063
abcb1a DMR17 (118 bp)	115	4	22322775	strongHyper	2.694	0.026	0.668 ± 0.052	0.601 ± 0.033
	154	4	22322814	strongHyper	2.371	0.043	0.223 ± 0.053	0.128 ± 0.083
	232	4	22322892	strongHyper	2.808	0.029	0.181 ± 0.062	0.105 ± 0.024

**Fig 4:**
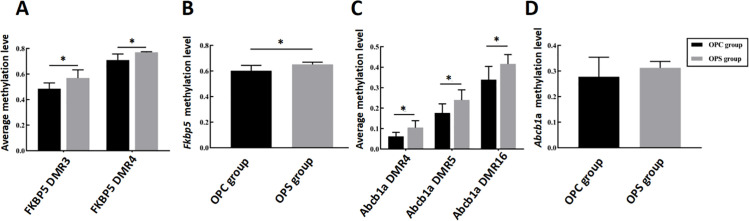
Effects of PS on DMRs methylation and total methylation levels of offspring placental GC barrier genes. **(A)** The methylation levels of DMRs and (B) the total methylation levels of FKBP5 were increased by PS on offspring. **(C)** The methylation levels of DMRs in ABCB1 were increased by PS on offspring, whereas (D) the total methylation levels of ABCB1 showed no significant change. Data are represented as mean ± SD (N = 6 per group). *Statistically significant difference compared with the OPC group (*P* < 0.05).

The results also revealed that there were three identical CpG methylation sites: abcb1a DMR4 at chr4:22182070, chr4:22181973, and abcb1a DMR5 at chr4:22182070. These sites were consistent with RRBS results when compared to those of the OPC group. Then, the CpG sites in each fragment were compared, and nine DMS were found to have the same methylation direction in both sequencing tests. There were 16 DMS in FKBP5, which showed different methylation statuses in the two tests, and the methylation directions of three DMS were the same. Furthermore, the FKBP5 gene exhibited significance and hypermethylation in both average methylation levels and total methylation levels, while the ABCB1 gene had significance and hypermethylation in average methylation levels but not in total methylation levels (Fig 4). Investigation revealed significant differences in methylation levels of ABCB1 and FKBP5 as indicated by RRBS and MethylTarget results. Additionally, these methylation sites were found to be distributed within the intron region, suggesting a potential impact of PS on the methylation level of both ABCB1 and FKBP5 introns.

### 3.6 PS significantly affects the transcription and expression of placental GC barrier-related genes

**Fig 5:**
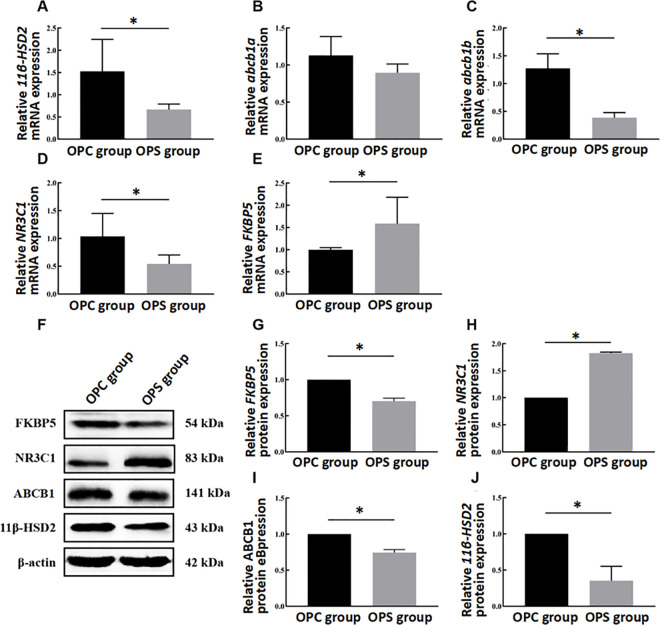
Effects of PS on mRNA and protein expression levels of placental GC barrier genes. **(A-D)** The mRNA levels of 11β-HSD2, ABCB1(abcb1a, abcb1b), NR3C1 and FKBP5. **(F)** Representative western blot images. **(G-J)** Relative protein levels of 11β-HSD2, ABCB1, NR3C1 and FKBP5. Data are represented as mean ± SD (N = 3 per group). *Statistically significant difference compared with the OPC group (*P* < 0.05).

To investigate the mRNA and protein expressions of placental GC barrier-related genes (11β-HSD2, ABCB1, NR3C1, and FKBP5), placental samples were collected from both the OPC and OPS groups. Significant reduced mRNA expression levels of placental *11*β-*HSD2* (*t (10)* =2.880, *P* = 0.016, Fig 5A) and ABCB1 (*t (10)* =7.874, *P* = 0.000, Fig 5C) were observed in the OPS group compared to the OPC group. However, there was no statistically significant difference in mRNA levels of ABCB1 between the OPS and OPC groups (*t (10)* =2.036, *P* = 0.081, Fig 5B). At the protein level, similar trends were observed for 11β-HSD2 (*t (4)* =5.667, *P* = 0.030, Fig 5F, J) and ABCB1 (*t (4)* =10.542, *P* = 0.009, Fig 5F, I). Furthermore, FKBP5 mRNA levels were significantly increased in the OPS group compared to the OPC group (*t (10)* =−2.428, *P* = 0.036, Fig 5E), while NR3C1 mRNA levels were downregulated (*t (10)* =2.729, *P* = 0.032, Fig 5D). Additionally, the protein expression level of NR3C1 was higher in the OPS group compared to the OPC group (*t (4)* =−73.099, *P* = 0.000, Fig 5F, I), whereas that of FKBP5 was lower (*t (4)* =12.061, *P* = 0.007, Fig 5F, G). These findings collectively suggest that maternal stress increases the corticosterone concentration in the offspring by weakening the placental GC barrier function.

## 4 Discussion

In this study, we utilized a chronic unpredictable mild stress (CUMS) pregnant rat model to investigate the relationship between prenatal stress (PS) and corticosterone, as well as its impact on DNA methylation of glucocorticoid (GC) barrier-related genes in the placenta. Here our results demonstrated that PS exposure significantly elevated plasma corticosterone levels in both pregnant rats and their offspring, while also regulating the expression of GC barrier-related genes through abnormal DNA methylation, including aberrant expression of DNMT and hypermethylation of ABCB1 and FKBP5. These findings provide preliminary evidence that PS disrupts the placental GC barrier through abnormal DNA methylation-mediated gene expression regulation, leading to increased corticosterone levels and affecting the growth and development of offspring.

Under stress or pathological conditions, high maternal concentrations of glucocorticoids (such as cortisol or corticosterone) may be transferred to the fetus through the placental barrier, thereby influencing fetal growth and development [32,33]. The observed elevation in corticosterone levels in both maternal and fetal circulation highlights the potential disruption of the placental barrier under CUMS conditions. This phenomenon suggests that stress may alter placental barrier function, increasing the transfer of maternal corticosterone to the fetus and ultimately raising offspring corticosterone levels, further confirming the critical role of the placental barrier in regulating maternal-fetal glucocorticoid balance [34,35].

Epigenetic changes are an important mechanism influencing the maternal placental barrier and are strong affected by environmental factors including stress [36]. Our study revealed that under PS conditions, the expression of methyltransferases in the placenta underwent significant changes, indicating that PS may influence gene methylation levels and sites [37,38]. Further research showed that the methylation levels of placental barrier-related genes 11β-HSD2, ABCB1, NR3C1, and FKBP5 varied, and their expression also changed significantly. Specifically, hypermethylation of abcb1a was negatively correlated with its mRNA expression, while hypermethylation of FKBP5 was positively correlated with its mRNA expression, possibly due to differences in their DMS and DMR. Notably, although no DNA methylation changes were observed for 11β-HSD2 and NR3C1, their expression still changed noticeably, suggesting that their mRNA expression may be regulated by factors such as transcription factors, histone modifications, and non-coding RNAs [38–40].

In terms of research methods, this study employed two DNA methylation detection techniques: Reduced Representation Bisulfite Sequencing (RRBS) and MethylTarget™ sequencing. RRBS provides broad coverage, enabling the detection of CpG sites across the entire genome, particularly in CpG islands and promoter regions, making it suitable for genome-wide methylation screening [29]. MethylTarget™ sequencing, on the other hand, offers high specificity and sensitivity, allowing for precise detection of methylation status in target genes or regions, making it ideal for targeted validation [30]. Leveraging the strengths of both methods, this study first used RRBS for preliminary genome-wide methylation screening to identify differentially methylated regions (DMRs) or sites (DMSs), followed by MethylTarget™ sequencing to validate the DMRs or DMSs of key genes identified by RRBS. This strategy not only enhanced the comprehensiveness of the detection but also ensured the reliability and accuracy of the results [30,31].

From a molecular mechanism perspective, increased DNA methylation levels can inhibit mRNA transcription, thereby affecting protein expression and the biological functions of genes in placental tissue [41,42]. abcb1a is expressed in placental trophoblast cells and can pump glucocorticoids and other substances from the fetal side back to the maternal side, reducing fetal exposure to high glucocorticoid levels [43]. FKBP5 regulates the activity of the glucocorticoid receptor (GR), influencing glucocorticoid signaling and thereby modulating fetal exposure to glucocorticoids [44]. 11β-HSD2 converts active glucocorticoids into inactive forms, reducing their passage through the placenta into the fetal circulation and protecting the fetus from high glucocorticoid exposure [45]. This study found that under PS stress conditions, the protein expression levels of these three genes were significantly lower than those in the control group, further confirming their importance in the placental barrier and their protective role in shielding the fetus from high glucocorticoid exposure. Additionally, since NR3C1 primarily encodes GR, which mediates the biological effects of glucocorticoids and regulates fetal development and metabolism [23,24], its increased expression in the placenta after PS exposure suggests that PS exacerbates the adverse effects of maternal high glucocorticoid levels on the placenta and fetus.

However, this study has some limitations. First, the relatively small number of rat placental samples (only 12) in the methylation study may have resulted in undetected methylation changes in 11β-HSD2 and NR3C1, limiting the reliability of the findings. Second, the molecular mechanisms by which PS affects DNA methylation in the placental barrier have not been fully elucidated, including the lack of glucocorticoid receptor expression level and behavioral assessments [46]. Furthermore, the study focused only on changes in offspring before and after birth and did not explore the long-term health effects of prenatal stress on offspring, such as metabolic diseases or neurobehavioral abnormalities in adulthood. Therefore, it is essential to expand the sample size in future studies to further investigate the effects of PS on DNA methylation of placental barrier-related genes and their underlying mechanisms, while also conducting relevant clinical research in pregnant women and newborns. Utilizing DNA methylation as a biomarker for prenatal stress (PS) can help predict the risk of developmental disorders in children and provide a scientific basis for developing personalized intervention strategies. At the same time, it can offer targeted prenatal care guidance for pregnant women, enabling timely identification and reduction of potential health risks [47]. Notably, with the rapid advancements in Network Medicine and AI technologies, these emerging tools will help uncover the significance of epigenetic modifications in the PS stress network of pregnant women, providing new perspectives for understanding the complex genetic mechanisms and developing rational therapeutic strategies [48,49].

## 5. Conclusions

Our results demonstrate that prenatal stress (PS) is associated with hypermethylation and abnormal expression of placental glucocorticoid (GC) related genes, including ABCB1 and FKBP5. These changes in methylation and expression might disrupt the integrity of placental GC barrier, leading to elevated corticosterone levels in offspring and impairing their growth and development. These findings suggest that DNA methylation of placental GC barrier genes may serve as a potential early warning marker for placental GC barrier dysfunction and increased susceptibility to multiple diseases in offspring.

## Supporting information

S1 Table Assessment of RRBS methylation results of placental GC barrier-related genes. (DOCX)

S1 Fig Schematic representation of the experimental procedure. (TIF)

S1 File Data set. (XLSX)
