# Supplementary material for: Prenatal stress increases corticosterone levels in offspring by impairing placental glucocorticoid barrier function
Source: PLoS One. 2025 Jul 18;20(7):e0313705. doi: 10.1371/journal.pone.0313705 (PMC12273964; doi:10.1371/journal.pone.0313705)
Supplement: S1 Table — (DOCX) [file pone.0313705.s001.docx]

Supplementary Material

**Tables S1 Assessment of RRBS methylation results of placental GC barrier-related genes**

| **Gene name** | **chromosome** | **position** | **Target name** |
| --- | --- | --- | --- |
| FKBP5 | 20 | 7975113 | FKBP5 DMR1 |
|  | 20 | 7975116 |  |
|  | 20 | 7977241 | FKBP5 DMR2 |
|  | 20 | 7977273 |  |
|  | 20 | 7978647 | FKBP5 DMR3 |
|  | 20 | 7991860 | FKBP5 DMR4 |
|  | 20 | 7991871 |  |
|  | 20 | 7992251 | FKBP5 DMR5 |
|  | 20 | 8000100 | FKBP5 DMR6 |
|  | 20 | 8002228 | FKBP5 DMR7 |
|  | 20 | 8019250 | FKBP5 DMR8 |
| abcb1a | 4 | 22140561 | abcb1a DMR1 |
|  | 4 | 22140572 |  |
|  | 4 | 22141030 | abcb1a DMR2 |
|  | 4 | 22141031 |  |
|  | 4 | 22141043 |  |
|  | 4 | 22141058 |  |
|  | 4 | 22141069 |  |
|  | 4 | 22141070 |  |
|  | 4 | 22165829 | abcb1a DMR3 |
|  | 4 | 22181973 | abcb1a DMR4 |
|  | 4 | 22181974 |  |
|  | 4 | 22182070 | abcb1a DMR5 |
|  | 4 | 22182101 |  |
|  | 4 | 22192599 | abcb1a DMR6 |
|  | 4 | 22222671 | abcb1a DMR7 |
|  | 4 | 22222675 |  |
|  | 4 | 22234557 | abcb1a DMR8 |
|  | 4 | 22234706 | abcb1a DMR9 |
|  | 4 | 22255279 | abcb1a DMR10 |
|  | 4 | 22279931 | abcb1a DMR11 |
|  | 4 | 22297010 | abcb1a DMR12 |
|  | 4 | 22297022 |  |
|  | 4 | 22304484 | abcb1a DMR13 |
|  | 4 | 22304577 |  |
|  | 4 | 22313828 | abcb1a DMR14 |
|  | 4 | 22320455 | abcb1a DMR15 |
|  | 4 | 22322568 | abcb1a DMR16 |
|  | 4 | 22322825 | abcb1a DMR17 |
|  | 4 | 22397453 | abcb1a DMR18 |
|  | 4 | 22397481 |  |
|  | 4 | 22397482 |  |
|  | 4 | 22397592 |  |
