# Supplementary figures and images for: Prenatal stress increases corticosterone levels in offspring by impairing placental glucocorticoid barrier function
Source: PLoS One. 2025 Jul 18;20(7):e0313705. doi: 10.1371/journal.pone.0313705 (PMC12273964; doi:10.1371/journal.pone.0313705)

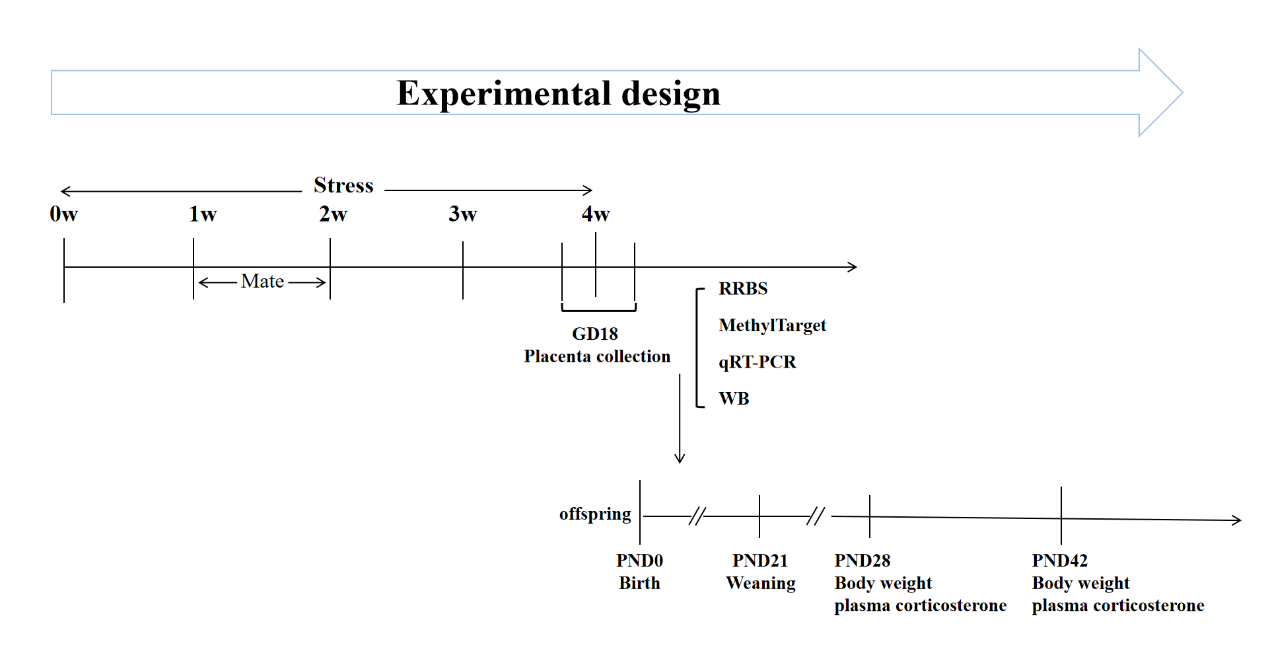

Supplement: S1 Fig — (TIF) [file pone.0313705.s002.tif]
